# Contribution to Herpesvirus Surveillance in Beaked Whales Stranded in the Canary Islands

**DOI:** 10.3390/ani11071923

**Published:** 2021-06-28

**Authors:** Idaira Felipe-Jiménez, Antonio Fernández, Marisa Andrada, Manuel Arbelo, Simone Segura-Göthlin, Ana Colom-Rivero, Eva Sierra

**Affiliations:** Atlantic Cetacean Research Center, Institute of Animal Health (IUSA), Veterinary School, University of Las Palmas de Gran Canaria (ULPGC), Trasmontaña s/n, Arucas, 35413 Las Palmas, Spain; idaira.felipe101@alu.ulpgc.es (I.F.-J.); antonio.fernandez@ulpgc.es (A.F.); manuel.arbelo@ulpgc.es (M.A.); siimone.andrea@gmail.com (S.S.-G.); acolomrivero@gmail.com (A.C.-R.); eva.sierra@ulpgc.es (E.S.)

**Keywords:** herpesvirus, alphaherpesvirus, DNA polymerase, PCR, beaked whale, *Ziphius cavirostris*, *Mesoplodon*, cetaceans

## Abstract

**Simple Summary:**

Herpesviruses (HVs) are a large family of DNA viruses infecting animals (including insects and mollusks) and humans. Cetaceans can be also infected by HVs presenting different range of lesions, from dermatitis to meningoencephalitis, or being asymptomatic. Several studies have addressed the question of HVs in cetaceans, although no previous systematic survey of HV in beaked whales (BWs) (*Ziphiidae* family) has been previously performed. The family *Ziphiidae*, which includes 22 species in 6 genera, is one of the most widespread families of cetaceans, with a strict oceanic habitat pattern. Beaked whales, Cuvier’s BW in particular, are one of the deepest diving whales and are of particular interest because of a notable relationship between military operations employing mid-frequency sonar and the mass stranding of BWs in different geographic areas, including the Canary Islands. In this study, we analyzed 55 BWs (294 samples) stranded in the Canary Islands from 1990 to 2017 by molecular methods (conventional nested polymerase chain reaction). Our results showed that 8 BWs were infected by HVs, although only three animals displayed lesions indicative of active viral replication. Phylogenetic analysis suggests that HV-BW sequences are species-specific, although more studies are needed to better address this question.

**Abstract:**

Herpesviruses (HVs) (*Alpha-* and *Gammaherpesvirinae* subfamilies) have been detected in several species of cetaceans with different pathological implications. However, available information on their presence in beaked whales (BWs) is still scarce. In this study, a total of 55 BWs (35 *Ziphius cavirostris* and 20 animals belonging to the *Mesoplodon* genus) were analyzed. Samples (*n* = 294) were obtained from BWs stranded along the coasts of the Canary Islands (1990–2017). Molecular detection of HV was performed by means of a conventional nested PCR based on the DNA polymerase gene. Herpesvirus was detected in 14.45% (8/55) of the analyzed BWs, including 2 positive animals from a previous survey. A percentage positivity of 8.57% was found within the Cuvier’s BW group, while the percentage of positivity rose to 25% within the *Mesoplodon* genus group (three *M. densirostris,* one *M. europaeus*, and one *M. bidens*). All the obtained sequences from this study belonged to the *Alphaherpesvirinae* subfamily, from which three are considered novel sequences, all of them within the *Mesoplodon* genus group. In addition, to our knowledge, this is the first description of HV infection in Gervais’ and Sowerby’s BWs. Three out of eight HV-positive BWs displayed histopathological lesions indicative of active viral replication.

## 1. Introduction

The family *Ziphiidae* is one of the most widespread families of cetaceans, with a strict oceanic habitat pattern. The Canary Islands, due to its volcanic origin and oceanic location, hold resident and transient populations of Beaked whales (BWs), with records of six different species. Beaked whales, Cuvier’s BW in particular, are one of the deepest diving whales. Depths of more than 2900 m and dive durations of over 2 h have been recently recorded in Cuviers’ BWs during single breath-hold dives [1]. Most of the distribution information of BWs is based on stranding records. They face threats from entanglement in fishing gear, ingestion of marine debris, and ship collision, among others [2,3,4]. They are also of particular interest because of a notable relationship between military operations employing mid-frequency sonar and the mass stranding of BWs in different geographic areas, including the Canary Islands [5,6,7,8]. Natural pathologies affecting these species include verminous arteritis by *Crassicauda* spp. [9], brucellosis [10,11] and virosis (morbillivirus and herpesvirus (HV)) [12,13,14,15,16,17,18]. The number of publications concerning HV infection in cetaceans has been increased in the past decades. However, very little information is available regarding the presence of these viruses in BWs.

The *Herpesvirales* order is split into three families: *Alloherpes-*, *Herpes-*, and *Malacoherpesviridae*. The *Herpesviridae* family is divided into three subfamilies (*Alpha-*, *Beta-*, and *Gammaherpesvirinae*), 13 genera, and 107 species [International Committee on Taxonomy of Viruses (ICTV) (https://talk.ictvonline.org/taxonomy/ (accessed on 3 September 2020)) [19]. A single, linear, double-stranded DNA characterizes these viruses, which can cause immunosuppression [20] and latent infections [21,22]. Moreover, HV can infect a wide range of hosts (mammals, birds, reptiles, amphibians, fish, mollusks, and insects) [23].

In cetaceans, several pathological findings associated with HV infection (alpha- and gamma-herpesvirus) have been documented, although in some cases, HV-related lesions may not be present in the infected animals [24]. Specifically, alphaherpesvirus has been related to fatal systemic infections [25], lymphoid necrosis [17], interstitial nephritis [18] and encephalitis and meningoencephalitis [26,27,28]. Gammaherpesvirus, however, has been described as mainly associated with mucocutaneous, skin, and genital lesions [16,29,30,31,32,33], although it has been recently described the first detection of gammaherpesvirus in the central nervous system of several striped dolphins (*Stenella coeruleoalba*) stranded in the Cantabrian Sea, Spain [34].

Herpesviruses have been found in many cetacean species, which are summarized in Table 1.

There are some documented cases of HV coinfection in cetaceans. Herpes- and morbillivirus co-infection have been reported in striped dolphins from the Mediterranean and Atlantic coasts [24,35,45] and from the Canary Islands [26]. Some cases of herpes- and papillomavirus coinfections have also been reported in Atlantic bottlenose dolphins from the Atlantic coast of the USA and Cuba [46,47].

To date, only seven HV-BW sequences, five alphaherpesviruses, and two gammaherpesviruses are available in GenBank. Concerning the alphaherpesviruses, most of them (3/5) have been detected in BWs stranded in the Canary Islands (two Cuvier’s BW (GU066291 and KY680659) and one Blainville’s BW (JN863234)). The only other BWs in which the alphaherpesvirus has been detected are three Cuvier’s BWs, two stranded in the Mediterranean Sea in 2012 (KP995682 and KP995685) and the other stranded on the Atlantic coast of the Spanish mainland in 2015 (KY680659).

The present study aims to detect HVs (novel or already known) in samples from stranded BWs in the Canary Islands and to correlate this positivity with histopathology to identify lesions compatible with the HV infection. In addition, phylogenetic relationship between the obtained sequences and all the available ones, within the *Herpesviridae* family, detected in cetaceans will be performed.

## 2. Materials and Methods

In this study, 294 samples from 55 BWs stranded along the coasts of the Canary Islands, from November 1999 to May 2017, were analyzed. These BWs included 35 Cuvier’s BWs and 20 specimens belonging to the *Mesoplodon* genus. This last group consists of 2 Sowerby’s BWs (*Mesoplodon bidens*), 7 Blainville’s BWs, 10 Gervais’ BWs (*Mesoplodon europaeus*), and 1 True’s BW (*Mesoplodon mirus*). Two of these animals have been already published previously (CET 243 and CET 294) [17,18]. Adults were more highly represented, while males and females were present in a similar proportion, in both groups of BWs. This information and other biological parameters (stranding epidemiology (type, location, and date) and life history data (species, age category, and sex)) are summarized in Table 2 and Table 3. Five codes of conservation condition were established [48]: Code 1 (extremely fresh carcass, as an animal that has recently died or euthanized), Code 2 (fresh carcass), Code 3 (moderate decomposition), Code 4 (advanced decomposition), and Code 5 (mummified or skeletal remains).

All the animals were submitted to a complete standardized necropsy [48,49], and representative tissue samples were collected for further analysis. For the histopathological study, collected samples were fixed in a 10% neutral buffered formalin solution, processed, and embedded in paraffin blocks, which were sectioned at 5 µm and stained with hematoxylin and eosin (HE). The slides were then visualized in an optical microscope with the objective to find HV-associated lesions. For molecular analysis, collected samples were frozen at −80 °C.

A wide range of tissue samples, according to availability in each case, were analyzed for the presence of HV DNA by PCR: lung (16.33%; (48/294)), kidney (15.65%; (46/294)), brain (12.93%; (38/294)), skin (12.59%, (37/294)), liver (12.24%; (36/294)), spleen (9.86%; (29/294)), mesenteric lymph node (8.16% (24/294)), skeletal muscle (6.12%; (18/294)), intestine (2.04%; (6/294)), prescapular lymph node (1.36%; (4/294)), mediastinal lymph node [0.68%; (2/294)], thyroid gland (0.34%; (1/294)), thymus (0.34%; (1/294)), palate (0.34%; (1/294)), and esophagus (0.34%; (1/294)). In addition, blood was analyzed in one animal (0.34%; (1/294)) (Table 2 and Table 3).

Thawed samples were mechanically macerated in a lysis buffer and centrifuged. DNA/RNA extraction was simultaneously carried out from each 300 μL macerated sample by pressure filtration, by means of a QuickGene R Mini 80 nucleic acid isolation instrument, with the DNA Tissue Kit S (QuickGene, Kurabo, Japan) according to the manufacturer’s instructions with some modifications: An RNA carrier (Applied BiosystemsTM, Thermo Fisher Scientific Waltham, MA, USA) was added during the lysis step, as previously published [50].

A panherpesvirus conventional nested polymerase chain reaction (PCR) was performed for HV detection, amplifying a fragment of the DNA polymerase gene of the *Herpesviridae* family of about 200 bp [51]. Two negative controls (non-template) for extraction and amplification and an amplification-positive control (known herpesvirus DNA previously obtained in our laboratory) were included in each protocol. Horizontal gel electrophoresis, in 5% agarose containing GelRed^®^ (Biotium, Inc. California, USA), was performed for 5 µL of the obtained amplicons from the second PCR. Purification of PCR products was carried out using a Real Clean spin kit (REAL^®^, Durviz, s.l.,Valencia, Spain) to perform sequencing (the Sanger method).

Furthermore, a reverse transcription real-time polymerase chain reaction (RT-qPCR) based on SYBRN^®^ Green dye (Bio-Rad Laboratories, Inc., California, CA, USA) [52] was performed for Cetacean morbillivirus (CeMV) detection only in those animals that were positive for HV.

The obtained HV sequences were compared with similar sequences retrieved from GenBank via a Blast search with the blastn algorithm (www.ncbi.nlm.nih.gov/blast/Blast.cgi/(accessed on 3 May 2021)) [53]. ClustalW was used to perform the HV multiple sequences alignment using MEGA X software (Pennsylvania, PA, USA) [54]. To construct the phylogenetic nucleotide tree, 73 alphaherpesvirus sequences were retrieved from GenBank. To root the phylogram, nine gammaherpesvirus sequences were used as the outgroup. The best substitution model for the nucleotide phylogenetic tree analysis was selected based on its lowest BIC score (Bayesian Information Criterion). Accordingly, a phylogenetic tree was constructed using the Maximum Likelihood Method and the Tamura 3-parameter with a discrete Gamma distribution to model the evolutionary rate differences among sites (5 categories (+G, parameter = 0.7779)). Bootstrap resampling (1000 replicates) was used to assess the reliability of the tree.

The nucleotide sequences were translated to the deduced amino acid sequences (70 alphaherpesvirus and 9 gammaherpesvirus sequences retrieved from GenBank). The deduced animo acid phylogenetic tree was built using the best substitution model based on its lowest BiC, a Maximum Likelihood Method and the Jones Taylor Thornton matrix-based model with a discrete Gamma distribution to model the evolutionary rate differences among sites (5 categories (+G, parameter = 17,401)). A bootstrap consensus tree for 1000 replicates was also performed.

A consensus tree was computed accepting the default 50% cut-off value (nodes supported by <50% of bootstrap replicates are collapsed), as previously proposed [55]. Only those bootstrap values equal or greater than 70% were considered valid among the remaining nodes (>50%). Furthermore, all sequences obtained from this study were submitted in GenBank, whose number accessions are from MZ066758 to MZ066765.

## 3. Results

Eight out of the fifty-five analyzed BWs (14.45%) had a positive result of the PCR test. Specifically, HV was detected in adult individuals of three *Ziphius cavirostris* and five animals belonging to the *Mesoplodon* genus (three *M. densirostris,* one *M. europaeus*, and one *M. bidens*), from which two had been previously published [17,18].

Moreover, these viruses were detected in 15 out of 294 (5.1%) analyzed samples. HV-positive samples were, by decreasing frequency, as follows: lungs (5/48, 10.41%), kidney (5/46, 10.89%), brain (2/38, 5.26%), liver (1/36, 2.77%), spleen (1/29, 3.44%), and prescapular lymph node (1/4, 25%).

### 3.1. Molecular Findings

**Figure 1:**
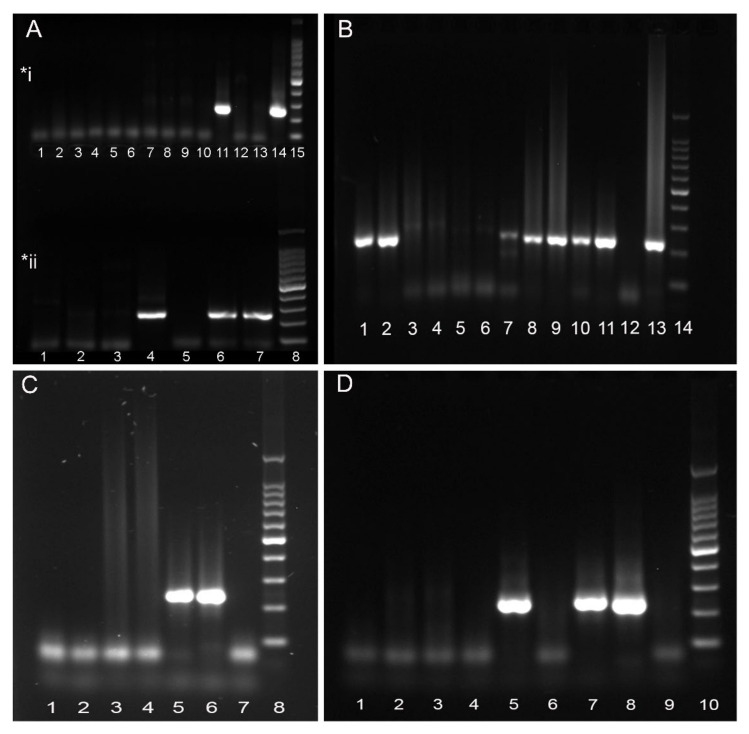
Herpesvirus-positive beaked whales results in agarose gel 5%, using nested conventional PCR. (**A**) (*i) Lane 11: Case 8 (CET 855—lung). Lane 14: PCR positive control. Lane 15: Molecular-weight size marker. (*ii) Lane 4: PCR positive control: Case 7 (CET 852—kidney). Lane 6 and 7: Case 5 (CET 771—brain). Lane 8: Molecular-weight size marker. (**B**) Lane 1: Case 4 (CET 379—lung). Lane 2: Case 4 (CET 379—kidney). Lane 8: Case 7 (CET 852—lung). Lane 9: Case 6 (CET 824—prescapular lymph node). Lane 10: Case 6 (CET 824—liver). Lane 11: Case 6 (CET 824—kidney). Lane 13: PCR positive control. Lane 14: Molecular-weight size marker. (**C**) Lane 5: Case 6 (CET 824—brain). Lane 6: PCR positive control. Lane 8: Molecular-weight size marker. (**D**) Lane 5: Case 2 (CET 259—kidney). Lane 7: Extraction positive control (CET 854—brain). Lane 8: PCR positive control/Case 7 (CET 852—kidney). Lane 10: Molecular-weight size marker. *Sequences from CET 243 and CET 294 have been already published.

Herpesvirus was detected in 3 out of 35 Cuvier’s BWs (8.6%). Specifically, Cases 3, 5, and 8 were positive for HV in one or more tissues (Table 4) (Figure 1A). Case 3 (CET 294), an adult female in advanced decomposition, showed positivity for HV in spleen and lung samples [17]. In Case 5 (CET 771), an adult female in good state of preservation (code 2), HV was found in brain tissue; in Case 8 (CET 855), an adult male in moderate decomposition, the positivity was found in the lung. Thus, a total of 4 samples out of 168 (2.4%) were positive for HV in the *Ziphius cavirostris* group. By decreasing frequency, HV was detected in lung (2/30, 6.6%), brain (1/21, 4.8%), and spleen (1/17, 5.9%) samples (Table 4). The size of the new sequences obtained in the present study, (Cases 5 and 8) ranged from 210 to 234 bp.

Five out of twenty animals (25%) were positive for HV within the *Mesoplodon* genus group (Cases 1, 2, 4, 6, and 7) in one or more tissues (Figure 1B–D). Specifically, a total of 11 samples out of 126 (8.7%) were positive for HV. By decreasing frequency, HV was detected in kidney (5/17, 29.4%), lung (3/18, 16.6%), liver (1/18, 5.5%), brain (1/17, 5.9%), and prescapular lymph node (1/1, 100%) samples (Table 4). HV was found in lung and kidney samples from Case 1 (CET 243), a very fresh (live stranded individual, which subsequently died) adult male of *M. densirostris* [18], Case 4 (CET 379), an adult male in code 2 of *M. bidens*, and Case 7 (CET 852), an adult female in code 2 of *M. densirostris*. Herpesvirus was detected in just one tissue (kidney) in Case 2 (CET 259), an adult female in code 2 of *M. europaeus*. In Case 6 (CET 824), an adult male in code 2 of *M. bidens*, HV was found in liver, prescapular lymph node, kidney, and brain tissues. The size from the obtained six new sequences from our study (Cases 2, 4, 6, and 7) ranged from 194 to 234 bp. None of the HV-infected animals were positive for CeMV. Phylogenetic analysis showed that all the sequences obtained from both groups of BWs belonged to the *Alphaherpesvirinae* subfamily.

#### 3.1.1. Nucleotide Identity

Eight sequences obtained from this study were new; two were previously published [17,18]. Nucleotide similarities are summarized in Table 5 and Table 6. The criteria to considerer a novel sequence are that the sequence are ≥100 bp long and has a <90% identity to the reference genome [56]. Based on this, the sequences from Cases 2 and 4 and the lung sample from Case 7 (7a) can be considered novel, as presented a 78.35%, 89.32% and 78.30%, similarity, respectively. Sequences from the kidney and lung in Case 1 (JN863234) were considered novel when they were published [18], although they currently show a high percentage of identity with a sequence detected in the brain of a striped dolphin stranded in the Mediterranean Sea in 2011. A novel sequence was obtained from the kidney in Case 2 (MZ066758); the sequence most closely related to this novel sequence was a previously published sequence detected in the prescapular lymph node of a Cuvier’s BW stranded in the Mediterranean Sea in 2012. Sequencing and further comparison with GenBank records showed a novel sequence highly related to cetacean alphaherpesvirus in the lung and spleen samples from Case 3 (GU066291) when they were published [17]. However, these sequences are currently identical to four previously described sequences in striped dolphins. A novel sequence was amplified from the lung and kidney in Case 4 (MZ066759), showing the highest similarity with sequences amplified from the same organs in Case 1 from our study. Two different sequences were obtained in Case 6, one sequence from the liver, prescapular lymph node, and kidney (6a) (MZ066761) and another from the brain (6b) (MZ066761). Two different sequences were also obtained from Case 7, one from the lung (7a) (MZ066763) and other from the kidney (7b) (MZ066764). Sequence 7a is considered novel.

**Figure 2:**
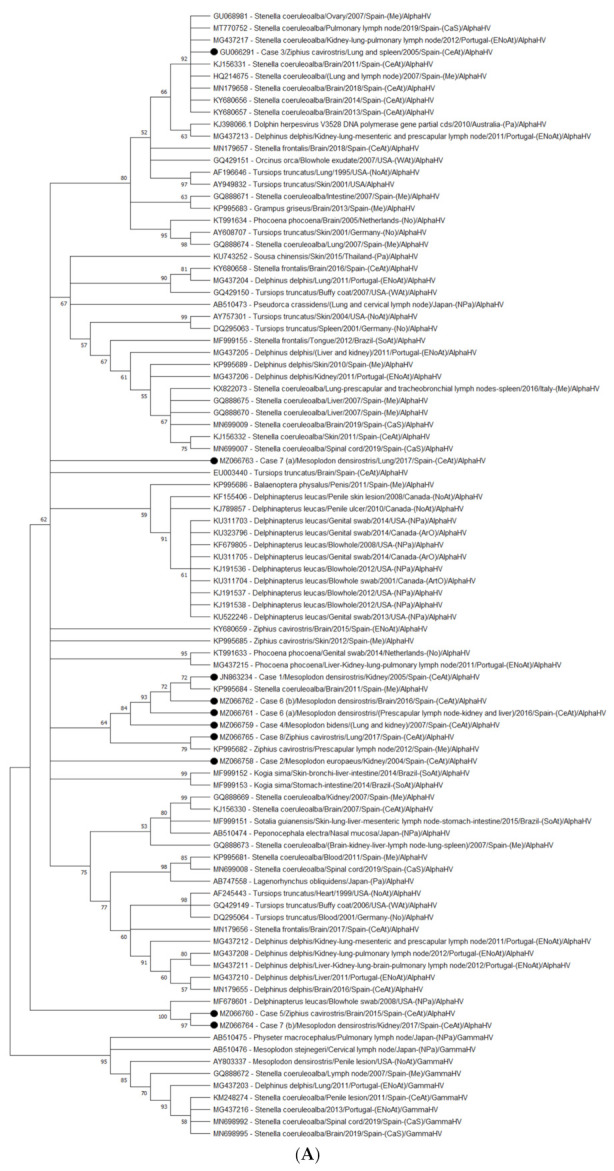

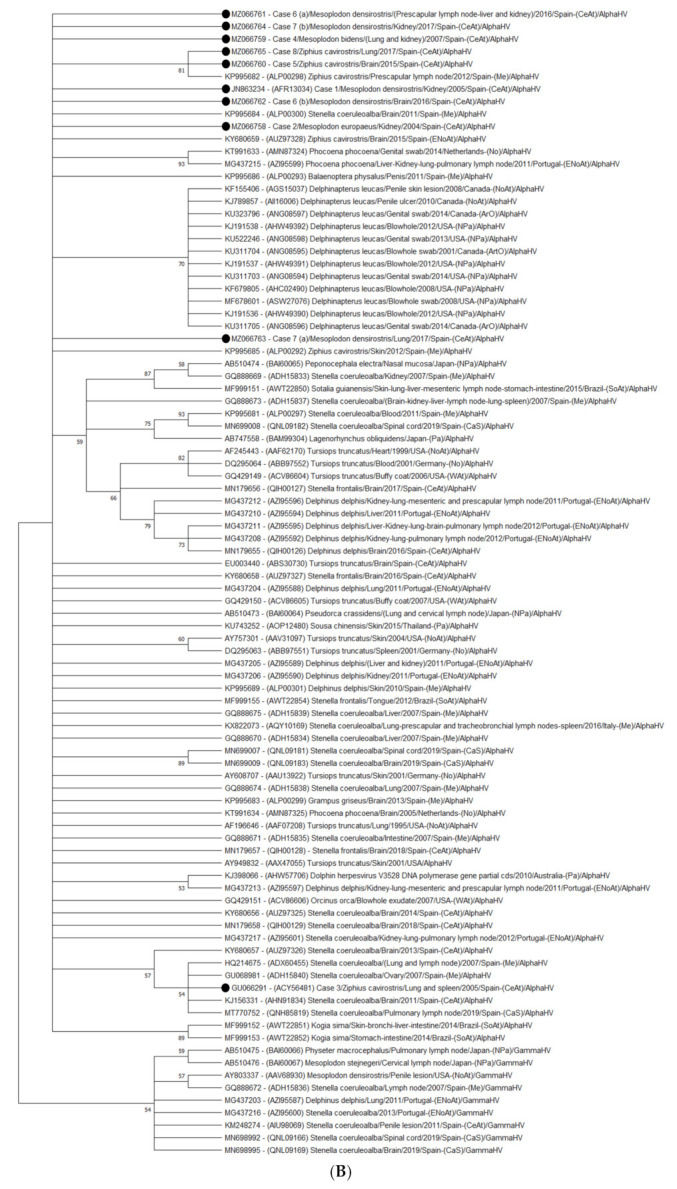
Maximum Likelihood phylogenetic trees. Nucleotide and amino acid sequences were identified with their corresponding accession number from GenBank, the host, the sample of detection, the date of collection, and the geographic area of stranding. Abbreviations: NoAt (North Atlantic Ocean); ENoAt (Northeast Atlantic Ocean); WAt (West Atlantic Ocean); CeAt (Central Atlantic Ocean); SoAt (South Atlantic Ocean); Me (Mediterranean Sea); CaS (Cantabrian Sea); Pa (Pacific Ocean); NPa (North Pacific Ocean); No (North Sea); ArO (Arctic Ocean). The bootstrap analysis was made to resample 1000 replicates and evaluate the reliability of the both trees. (**A**) Molecular phylogenetic analysis based on 94 nucleotide sequences from the polymerase gene of cetacean alphaherpesvirus. The Neighbor-Join and BioNJ algorithms along with the Tamura 3-parameter model and Gamma distribution were used to construct the tree. (**B**) Molecular phylogenetic analysis based on 92 amino acid sequences from the polymerase gene of cetacean alphaherpesviruses. The Neighbor-Join and BioNJ algorithms along with the JTT model and Gamma distribution were used to construct the tree.

The nucleotide phylogenetic analysis (Figure 2A) showed that sequences from Cases 1 (kidney), 4 (lung and kidney), 6a (liver, prescapular lymph node, and kidney), 6b (brain), and 8 (lung) clustered together in a clade supported by a bootstrap value of 64, with two subclades: one containing sequences from Cases 1, 4, 6a, and 6b and a sequence from a striped dolphin stranded in the Mediterranean Sea in 2011 (KP995684) (84 bootstrap value) and another containing two sequences, one from Case 8 and another from a Cuvier’s BW stranded in the Mediterranean Sea in 2012 (KP995682) (79 bootstrap value).

The sequence from Case 7 (lung) did not cluster with any sequence within the nucleotide phylogenetic tree, nor did the sequence from Case 2 (kidney), even if both of them belong to a large clade (62 bootstrap value) containing all the cetaceans alphaherpesvirus published until now.

However, the sequence from Case 3 (lung and spleen) clustered with sequences detected in striped dolphins stranded in the Mediterranean and Atlantic coasts (92 bootstrap value); sequences from Cases 5 (brain) and 7 (kidney) clustered together (97 bootstrap value) and with a sequence (blowhole swab) from a beluga whale (MF678601) (100 bootstrap value).

#### 3.1.2. Amino Acid Identity

Blast analyses of translated amino acid sequences showed similar results to those of the nucleotides. However, some differences were observed for Cases 1, 4, 6a, and 7a. Case 1 showed the highest similarity (79.13%, 100 QC) with a sequence detected in a beluga whale (ANG08598). Sequences from the lung and kidney in Case 4 were very similar (95.59%, 87% QC) to a sequence detected in the brain of a striped dolphin stranded in the Mediterranean Sea in 2011 (ALP00300), and to a sequence detected in a Cuvier’s BW stranded in the Mediterranean Sea in 2012 (ALP00298) (94.12%, 87% QC). The sequence from the prescapular lymph node, liver, and kidney in Case 6 (6a) was very similar (97.06%, 99 QC) to sequence ALP00300. The lung sequence from Case 7 showed the highest similarity (72.73%, 94% QC) with a sequence detected in the skin of a Cuvier’s BW stranded in the Mediterranean Sea in 2012 (ALP00292). Amino acid similarities are summarized in Table 5 and Table 6.

Phylogenetic analysis (Figure 2B) showed that the tree based on deduced amino acids consists of 45 alphaherpesvirus branches and a root that contains nine gammaherpesvirus sequences. All the obtained sequences from our study take part of a large polyphyletic clade within which seven sequences (Cases 1, 2, 4, 6a, 6b, 7a, and 7b) did not form sub-clades with any sequences of any of the previously identified HV in cetaceans; while sequences from Case 5 (brain) and Case 8 (lung) clustered together with a sequence obtained from the prescapular lymph node of a Cuvier’s BW stranded in 2012 in the Mediterranean Sea (ALP00298) (bootstrap value of 81). Finally, the sequence from Case 3 clustered together with sequences obtained from striped dolphins stranded in the Mediterranean Sea and the Atlantic coasts of Spain (mainland and Canary Islands) and the Cantabrian Sea (54 bootstrap values).

### 3.2. Gross and Histopathological Findings

None of the HV-positive animals showed gross lesions associated with Herpesvirus infection, except Case 7, which presented several ulcerative and well-defined round skin lesions.

**Figure 3:**
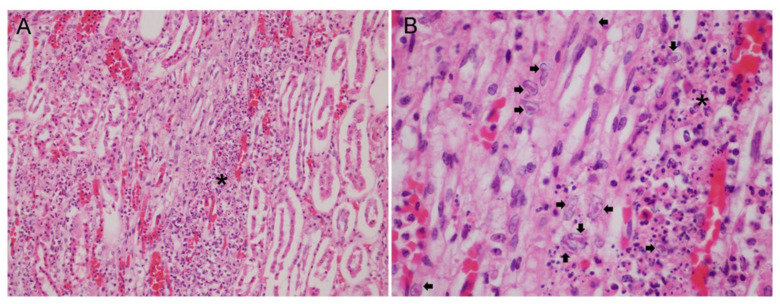
Histopathological findings from the kidney in Case 8 (CET 852). (**A**) Moderate and multifocal acute tubular necrosis in the medullary zone (asterisk). Hematoxylin and eosin, X20. (**B**) A moderate and multifocal presence of intranuclear inclusion bodies (arrows) associated to acute tubular necrosis (asterisk). Hematoxylin and eosin, X60.

At the histopathological level, three animals presented lesions attributable to HV infection, one within the *Ziphius cavirostris* group and two within the *Mesoplodon* genus group. Specifically, Case 3 (*Z. cavirostris*) presented diffuse lymphoid and splenic necrosis with intranuclear inclusion bodies in monocytes [17]; Case 1 (*M. densirostris*) [18] and Case 7 (*M. densirostris*) displayed similar lesions, characterized by membranous glomerulonephritis and lymphoplasmacytic interstitial nephritis at the cortico-medullary region, moderate multifocal interstitial and tubuloepithelial necrosis with the presence of intranuclear inclusion bodies within tubuloepithelial cells in the renal medulla; mainly in the blood capillary network defined as the vasa recta of the kidney (*vasa rectae renis*) (Figure 3).

Additional findings in the other positive HV-BWs were as follows: mild multifocal parasitic bronchopneumonia with the presence of intraluminal nematodes in Cases 1 and 4; generalized lymphadenopathy and lymphoplasmacytic interstitial nephritis in Case 4; severe parasitic nephritis and verminous mesenteric arteritis by *Crassicauda* sp., a moderate multifocal suppurative bronchopneumonia, and moderate multifocal suppurative lymphadenitis in Case 5; periductal fibrosis with lymphoplasmacytic pericolangitis in the liver, as a result of severe parasitic infection, in Case 6; the presence of severe hyaline membranes and mild multifocal interstitial bronchopneumonia in Case 8.

In addition, Case 2 also displayed several skin lacerations in the caudal peduncle produced by fishing tackles and diffuse congestion and hemorrhages in the lungs, liver, kidneys, and adrenal glands, and Case 6 showed active stranding related lesions, consistent with skin lacerations, a hypercontraction of muscle fibers, and hyaline globules within hepatocyte cells.

## 4. Discussion

This study represents the first systematic survey of HV infection in cetaceans from the *Ziphiidae* family. Beaked whales are found in all oceans and are of particular interest because they are one of the deepest diving whales and there is a proven relationship between several mass stranding events of BWs and military operations employing mid-frequency sonar [1,7,8].

We have detected HV in 14.45% (*n* = 8) of the surveyed BWs (*n* = 55). A percentage positivity of 8.57% (3/35) was found within Cuvier’s BW group, while the percentage of positivity rose to 25% (5/20) within the *Mesoplodon* group. Previously published prevalence of HV infection in stranded cetaceans include: 5.3% in the Western North Pacific (Japan) [40], 3.7% in Brazil [44], 7.8% in Portugal [35], and 41.2% in Cantabria, Spain [34]. Most of the references of HV infection in BWs are case reports: two alphaherpesviruses in a Cuvier’s BW and a Blainville’s BW stranded in the Canary Islands [17,18] and one gammaherpesvirus in a Blainville’s BW stranded in the USA [16]. The detection of a gammaherpesvirus in a Stejneger’s BW from the Japanese coasts took part of a survey of 76 stranded cetaceans that include 4 BWs (one Stejneger’s BW and three Blainville’s BWs) [40]. In addition, three sequences from the brain, skin, and prescapular lymph nodes of Cuvier’s BWs are available in GenBank (KY680659, KP995685, and KP995682).

All the sequences obtained from this study belonged to the *Alphaherpesvirinae* subfamily in contrast to similar previous studies, in which both alpha and gammaherpesvirus were detected [27,34,35,40,44].

The *Ziphiidae* includes 22 species in 6 genera, being the second largest family of cetaceans after the *Delphinidae* [57]. Herpesvirus sequences detected in BWs from our study should be then analyzed by species. Specifically, sequences from Cuvier’s BWs are not novel sequences, displaying higher homologies (100–98.06%) with sequences from striped dolphins stranded in the Mediterranean Sea and central and northeast Atlantic coasts (the Canary Islands and Portugal) (MG437217, KY680657, KY680656, and KJ156331) [26,34,35] (Case 1) and with a sequence detected in other Cuvier’s BW from the Mediterranean Sea (KP995682) (Cases 5 and 8). The sequence KP995682 can be considered novel since it shows the highest homology (83.98%) with the sequence detected in the brain of a striped dolphin stranded in the Mediterranean Sea in 2011 (KP995684), which in turn showed the highest homology with the sequence from Case 1 in our study (95.5%) and with the sequence KP995682 (83.98%). This relationship could indicate that HV transmission has occurred between these proximal regions, as previously suggested for other alphaherpesviruses [26,35,44] and between these two different species. Cuvier’s BWs are found in most oceans and seas worldwide (in temperate, subtropical, and tropical waters), and have the most extensive range of all BWs species, although the seasonality and migration patterns of this species are still unknown [58].

Regarding the *Mesoplodon genus*, sequences from the *M. densirostris* species are, in general, highly (97.4–93%) related to a sequence detected in the brain of a striped dolphin stranded in the Mediterranean Sea in 2011 (KP995684) (Cases 1, 6b, and 7b). As we mentioned before, this sequence, even if detected in a striped dolphin, showed its highest homologies with sequences detected in members of the *Ziphiidae* family, suggesting the idea of HV transmission from BWs to the striped dolphin species rather than the contrary. The only sequence from Case 7a is considered novel within the *M. densirostris* species in our study, being related to the sequence obtained from the penile skin lesion of a beluga whale stranded in St. Lawrence Estuary (Canada) (KF155406). This beluga sequence was also considered novel and tentatively named beluga whale herpesvirus [36]. The relationship (geographical and interspecies) between these two sequences is, for the moment, unknown. Blainville’s BWs are little-known members of the *Ziphiidae*, living in tropical to temperate waters worldwide. There is little information on the abundance of Blainville’s BWs worldwide, although they are considered to have the most extensive distribution of any whale in the *Mesoplodon* genus [59].

Two novel sequences were obtained from *M. europaeus* (Case 2) and *M. bidens* (Case 4) species. Sequence from Case 2 was related to sequence KP995682, detected in a Cuvier’s BW from the Mediterranean Sea; as most of the Cuvier’s BWs from our study; while sequence from Case 4 was related to sequence from Case 1 (JN863234), detected in a Blainville’s BW stranded in the Canaries and taking part of this study. Interspecific interactions between these four species of BWs could explain HV transmission within the *Ziphiidae* family. Gervais’s and Sowerby’s BWs are little-known members of the *Ziphiidae*. Both species of BWs are distributed throughout the Atlantic Ocean, although it is unknown if they undertake seasonal movements or migrations [60,61]. The presence of both BWs species in the Canary archipelago is reported as sporadic [62,63].

Regarding the nucleotide phylogeny of the reported HV-BW sequences from our study (*n* = 10), most of the sequences (80%, 8/10) clustered with a sequence previously identified in a Cuvier’s BW, but also from other species, such as striped dolphins or beluga whales. Specifically, the sequence from the lung in Case 7 (7a) clustered with the sequence from Case 5 and with a sequence detected in the blowhole swab of a beluga whale (MF678601) in the nucleotide tree. The phylogenetic analyses showed that the virus isolated from this beluga whale grouped with members of the genus *Varicellovirus*, in the subfamily *Alphaherpesvirinae*, and it was tentatively named Monodontid alphaherpesvirus 1 [64]. Thus, although most of the HV-BWs sequences from our study are quite host-specific, as previously suggested for the members of *Herpesviridae* [65], it seems that there is a possible interspecific transfer of these viruses. The obtained results from the amino acid phylogenetic analysis showed, however, that sequences from animals within the *Mesoplodon* genus group (70%, 7/10) did not cluster with any previously identified sequence. This discrepancy between trees could be due to the presence of too few characters in the aminoacid dataset, being the phylogenetic signal for the tree reconstruction, in accordance, too small. Sequences from the Cuvier’s BW group (*n* = 3) clustered with a sequence previously detected in a Cuvier’s BW (20%, 2/10) or a striped dolphin (10%, 1/10). However, apart from this study, there are no alphaherpesvirus sequences from animals within the *Mesoplodon* genus available in GenBank. More and larger sequences will be needed to better understand the species specificity of HV-BWs. In conclusion, from the phylogeny analyses, it can be observed that the obtained BW sequences from this study are more often closely related to each other and occasionally with sequences from other cetacean species, specifically striped dolphins and beluga whales. However, the short partial sequences of the catalytic subunit (UL30) of the DNA polymerase available from this and other studies, only allow for subfamily identification [66]. Further studies are needed to better understand the phylogenetic relationship between these and other BW sequences within these species.

In addition, to our knowledge, this is the first report of HV infection in two species of BWs: Gervais’ and Sowerby’s BWs, respectively.

Two different sequences were obtained within the same animal in Cases 6 and 7. Coinfection with different viral strains seems to be a common feature of HV infection in cetaceans as it has been previously reported in several studies [24,26,30,35].

Most HV-BWs in our study were detected in the lung and/or kidney, representing a percentage of 21.3% considering all the analyzed samples. However, if we consider only positive samples, this percentage increased to 66.7%. Moreover, HV was detected in the lung in 62% of the positive BWs (5/8) and the kidney in the same proportion. However, if we consider the two groups of BWs, HV was detected in the kidney in 100% of HV-positive animals within the *Mesoplodon* group of our study. A minor proportion of samples were positive for HV, specifically the brain, prescapular lymph node, liver, and spleen, being the first identification of an HV DNA sequence in a liver sample from a BW.

The capacity of HV to cause disease is uncertain displaying the broad pathogenic and epidemiological features of the disease. In cetaceans, there have been descriptions from asymptomatic cases of HV infections to systemic and/or central nervous system infections. No gross or histopathological lesions attributable to the virus infection were observed in most of the positive samples in our study (62.5%). In addition, despite the presence of skin lesions compatible with herpesvirus infection in Case 7, no HV DNA was detected in the corresponding sample. All the HV-infected BWs from our study were adults, although only three out of eight (37.5%) displayed histopathological lesions indicative of active herpesviral replication, consistent with previous publications [17,18]. It is well known that the role of the viral factors in the course of a infection is both determined by viral and host factors, including the immune status itself or in combination with the age of the individuals [67,68]. In this study, we described multifocal interstitial nephritis, tubuloepithelial necrosis, and the presence of several intranuclear inclusions in Case 7, lesions that are very similar to those previously described in other *M. densirostris* stranded in the Canary Islands 13 years earlier [18]. The animal from Case 3 (CET 294) presented an advance stage of decomposition (code 4), which could partially impair DNA integrity for PCR detection, as previously published [35]. However, HV was detected in lung and spleen samples, which showed severe histopathological lesions and large number of intranuclear inclusion bodies indicative of early stages of the infection and high viral load, allowing molecular detection and identification of the virus. In a similar way, HVs were detected in code 4 cetaceans, specifically two mysticeti species stranded in the Mediterranean Sea [42] and in an Atlantic spotted dolphin stranded in Brazil [44].

## 5. Conclusions

This research describes the presence of HV in BWs stranded in the Canary Islands over a 19-year period (1999–2017) by molecular methods. Our results showed a prevalence of positive BWs of 14.45% (8/55), representing the first systematic survey of this pathogen in BWs. Three out of eight HV-positive BWs displayed histopathological lesions indicative of active viral replication, which is in concordance with the latent period of most herpesviruses. However, HVs are also capable of causing severe disease in association with other pathogens, such as CeMV. No CeMV infection was detected in any of the HV-positive BWs, highlighting the potential disease-causing capacity of these viruses as primary pathogens. Eight new HV sequences were detected in this study, which were analyzed and compared to all HV existing sequences in cetaceans. Most of these sequences did not cluster with any other sequences in the amino acid phylogenetic trees, indicating a possible species-specificity in BWs; although testing for clustering and host specificity would need more tailored analyses. In addition, three novel sequences of a partial fragment of the conserved DNA polymerase of HVs are described, all of them within the *Mesoplodon* genus group. To our knowledge, this work is the first to describe herpesvirus infection in two species of BWs: Gervais’ and Sowerby’s BWs.

## Figures and Tables

**Table 1:** Summary of reported herpesvirus infection in cetaceans worldwide.

Species	Locality	AlphaHV	GammaHV
*Phocoena phocoena*	Sweden, Netherlands, Portugal Northern Hemisphere	[27,28,35]	[27,32]
*Delphinapterus leucas*	Canada	[36,37]	-
*Tursiops truncatus*	Atlantic coast of United States of America (USA) Spain: The Canary Islands Mediterranean Sea	[25,30,38]	[29,30,39]
*Pseudorca crassidens*	Pacific waters	[40]	-
*Peponocephala electra*	Pacific waters	[40]	-
*Lagenorhynchus obliquidens*	Pacific waters	[41]	-
*Balaenoptera physalus*	Mediterranean Sea	[42]	-
*Stenella coeruleoalba*	Portugal Spain: The Canary Islands Cantabrian Sea	[34,35]	[31,34,35]
*Delphinus delphis*	Portugal	[35]	[35]
*Lagenorhynchus obscurus*	South America	[43]	-
*Stenella frontalis*	South America	[44]	-
*Sotalia guianensis*	South America	[44]	[33]
*Kogia sima*	South America	[44]	-
*Ziphius cavirostris*	Spain: The Canary Islands	[17]	-
*Mesoplodon densirostris*	Spain: The Canary Islands Atlantic coast of United States of America (USA)	[18]	[16,30]
*Grampus griseus*	Atlantic coast of United States of America (USA)	-	[30]
*Physeter macrocephalus*	Japanese coast	-	[40]
*Balaenoptera acutorostrata*	Mediterranean Sea	-	[42]
*Inia boliviensis*	South America	-	[44]
*Mesoplodon stejnegeri*	Japanese coast	-	[40]

Note: (-): Non reported cases.

**Table 2:** Biological, stranding conditions and analyzed samples for herpesvirus detection of the 35 *Ziphius cavirostris* specimens included in the present study.

ID CODE	SEX	AGE	SD	SL	SS	DC	TESTED SAMPLES
CET 86	F	A	27/11/1999	Tenerife	A	3	Skin, lung, liver, kidney
CET 103	M	J	19/04/2000	Fuerteventura	D	3	Lung, liver, kidney, brain
CET 108	F	A	10/06/2000	Tenerife	D	3	Skin, skeletal muscle, lung, liver, kidney
CET 113	F	S	16/07/2000	Tenerife	D	3	Skin, skeletal muscle
CET 181	M	S	24/09/2002	Fuerteventura	A	2	Skin, skeletal muscle, lung, mediastinal and mesenteric lymph node, liver, kidney, brain, spleen
CET 182	M	S	24/09/2002	Fuerteventura	D	2	Skin, skeletal muscle, lung, liver, mesenteric lymph node, kidney, brain, spleen
CET 183	M	S	24/09/2002	Fuerteventura	D	2	Skin, skeletal muscle, liver, mesenteric lymph node, kidney, brain
CET 184	M	S	24/09/2002	Fuerteventura	D	2	Skin, skeletal muscle, lung, liver, mediastinal and mesenteric lymph node, kidney, brain, spleen, thyroid
CET 189	F	A	27/09/2002	Fuerteventura	D	4	Skin, lung, liver, kidney
CET 236	F	C	21/03/2004	La Graciosa	D	3	Skin, skeletal muscle, lung, liver, kidney, brain
CET 264	F	N.D.	23/07/2004	Fuerteventura	D	4	Liver, skeletal muscle, lung, kidney
CET 265	M	A	24/07/2004	Fuerteventura	D	4	Skin, skeletal muscle, lung, liver, kidney
* CET 294	F	A	18/04/2005	Fuerteventura	D	4	Skin, skeletal muscle, lung, liver, spleen
CET 304	F	C	13/07/2005	Fuerteventura	D	2	Skin, skeletal muscle, lung, liver, kidney
CET 322	M	A	17/02/2006	Gran Canaria	D	4	Skin, lung, liver, kidney
CET 352	N.D.	J	06/07/2006	Tenerife	D	3	Lung, kidney, brain, spleen
CET 471	F	S	06/11/2008	Fuerteventura	D	2	Lung, kidney, brain, spleen
CET 503	F	A	21/09/2009	Gran Canaria	D	4	Lung, kidney
CET 576	F	A	16/05/2011	Lanzarote	D	2	Lung, kidney, brain, spleen
CET 579	M	S	13/06/2011	Tenerife	D	4	Lung, mesenteric lymph node, kidney, brain, spleen
CET 591	F	A	01/11/2011	Tenerife	D	4	Lung, prescapular lymph node, kidney, brain, spleen
CET 593	M	A	18/11/2011	Gran Canaria	D	4	Skin, lung, prescapular lymph node, liver, kidney
CET 620	M	A	20/05/2012	Gran Canaria	D	4	Skin, lung, liver, kidney, spleen
CET 624	F	A	13/07/2012	La Graciosa	D	3	Skin, lung, liver, mesenteric lymph node, kidney, brain
CET 645	M	J	09/02/2013	Lanzarote	D	4	Skin, liver, brain
CET 680	F	N	02/07/2013	Gran Canaria	D	4	Lung, intestine, mesenteric lymph node, kidney, brain, spleen
CET 688	F	A	18/11/2013	Gran Canaria	D	4	Brain
CET 712	F	S	28/04/2014	Fuerteventura	D	4	Prescapular lymph node, spleen
CET 719	F	A	06/06/2014	Lanzarote	D	3	Lung, mesenteric lymph node, kidney, spleen
CET 720	N.D.	S	10/06/2014	Fuerteventura	D	4	Lung, mesenteric lymph node, kidney, brain
CET 770	M	S	28/07/2015	Tenerife	D	3	Lung, intestine, mesenteric lymph node, brain, spleen
CET 771	F	A	05/08/2015	Tenerife	D	2	Lung, intestine, mesenteric lymph node, kidney, brain, spleen
CET 818	M	S	16/08/2016	Gran Canaria	D	4	Lung, intestine, mesenteric lymph node, kidney, brain, spleen
CET 833	N.D.	N.D.	13/02/2017	Tenerife	D	4	Lung, mesenteric lymph node, kidney, brain
CET 855	M	A	22/05/2017	Gran Canaria	D	3	Lung, intestine, mesenteric lymph node, kidney, brain, spleen

Remarks: (*): animal previously published; SD (stranding date); SL (stranding location); SS (stranding stage: A = alive; D = dead); DC (decomposition stage); SEX (F = female, M = male, ND = not determined); AGE (A = adult; S = subadult; J = juvenile; C = calf, N = neonate). DC (1 = extremely fresh carcass; 2 = fresh carcass, 3 = moderate decomposition, 4 = advanced decomposition, and 5 = mummified or skeletal remains).

**Table 3:** Biological, stranding conditions and analyzed samples for herpesvirus detection of the 20 animals belonging to the *Mesoplodon* genus included in the present study.

ID CODE	SPECIES	SEX	AGE	SD	SL	SS	DC	TESTED SAMPLES
CET 134	*M. europaeus*	F	C	28/06/2001	Gran Canaria	A	1	Skin, lung, liver, kidney, brain
CET 180	*M. densirostris*	F	A	24/09/2002	Fuerteventura	A	2	Skin
CET 185	*M. europaeus*	F	A	24/09/2002	Fuerteventura	D	2	Skin, skeletal muscle, lung, liver, mesenteric lymph node, kidney, brain, spleen
CET 213	*M. densirostris*	F	A	28/06/2003	Gran Canaria	A	1	Skin, skeletal muscle, lung, liver, kidney, brain
* CET 243	*M. densirostris*	M	A	18/04/2004	Tenerife	A	1	Skin, skeletal muscle, lung, liver, kidney
CET 259	*M. europaeus*	F	J	21/06/2004	Fuerteventura	D	2	Skin, skeletal muscle, lung, liver, kidney, brain
CET 333	*M. europaeus*	F	S	28/03/2006	El Hierro	A	2	Skin, skeletal muscle, lung, thymus, liver, mesenteric lymph node, kidney, brain, spleen
CET 334	*M. europaeus*	F	S	28/03/2006	El Hierro	A	2	Skin, skeletal muscle, lung, liver, kidney, brain, spleen
CET 338	*M. europaeus*	F	J	06/04/2006	Gran Canaria	D	2	Skin, skeletal muscle, lung, liver, blood, mesenteric lymph node, kidney, brain, spleen
CET 354	*M. europaeus*	M	C	28/07/2006	Tenerife	D	4	Skin, lung, liver, kidney, spleen
CET 379	*M. bidens*	M	A	16/04/2007	Lanzarote	D	2	Skin, lung, liver, kidney, brain, spleen
CET 510	*M. europaeus*	M	A	14/12/2009	Lanzarote	D	2	Skin, lung, liver, mesenteric lymph node, kidney, brain
CET 547	*M. europaeus*	M	A	29/08/2010	Fuerteventura	D	4	Skin, lung, liver, mesenteric lymph node, kidney, brain
CET 631	*M. europaeus*	M	A	21/10/2012	Fuerteventura	D	4	Skin, lung, penis, palate, esophagus, brain
CET 636	*M. mirus*	M	S	30/11/2012	El Hierro	D	2	Skin, lung, liver, mesenteric lymph node, kidney, brain, spleen
CET 695	*M. densirostris*	F	A	12/07/2014	Lanzarote	D	4	Skin, lung, liver, mesenteric lymph node, kidney, brain, spleen
CET 711	*M. densirostris*	M	S	03/04/2014	El Hierro	D	5	Skin, lung, liver, mesenteric lymph node, kidney, brain, spleen
CET 824	*M. densirostris*	F	A	11/11/2016	Fuerteventura	D	2	Skin, prescapular lymph node, liver, kidney, brain, spleen
CET 827	*M. bidens*	F	A	07/12/2016	La Gomera	A	4	Skin, lung, liver, mesenteric lymph node, intestine, brain, spleen
CET 852	*M. densirostris*	F	A	05/05/2017	Fuerteventura	D	2	Skin, lung, liver, mesenteric lymph node, kidney, brain, spleen

Notes: (*): animal previously published; SD (stranding date); SL (stranding location); SS (stranding stage: A = alive; D = dead); DC (decomposition stage); SEX (F = female, M = male), AGE (A = adult, S = subadult; J = juvenile; C = calf); SS (stranding stage: A = alive, D = dead); DC (1 = extremely fresh carcass, 2 = fresh carcass, 3 = moderate decomposition, 4 = advanced decomposition, and 5 = mummified or skeletal remains).

**Table 4:** Beaked whales and samples positive for herpesvirus in our study.

CASE *n*°	ID CODE	SPECIES	SEX	AGE	SD	SL	SS	DC	HV-POSITIVE SAMPLES	HV-OBTAINED SEQUENCES
1	* CET 243	*M. de*	M	A	18/04/2002	T	A	1	Lung, kidney	(Arbelo et al., 2012)
2	CET 259	*M. eu*	F	A	21/06/2004	F	D	2	Kidney	MZ066758
3	* CET 294	*Z. ca*	F	A	18/04/2005	F	D	4	Lung, spleen	(Arbelo et al., 2010)
4	CET 379	*M. bi*	M	A	16/04/2007	L	D	2	Lung, kidney	MZ066759
5	CET 771	*Z. ca*	F	A	06/08/2015	T	D	2	Brain	MZ066760
6	CET 824	*M. de*	F	A	11/11/2016	F	D	2	(a) Liver, prescapular lymph node, kidney; (b) brain	(a) MZ066761, (b) MZ066762
7	CET 852	*M. de*	F	A	05/05/2017	F	D	2	(a) Lung; (b) kidney	(a) MZ066763, (b) MZ066764
8	CET 855	*Z. ca*	M	A	22/05/2017	GC	D	3	Lung	MZ066765

Remarks: (*): animal previously published. *M. de* (*Mesoplodon densirostris*); *M. eu* (*Mesoplodon europaeus*); *Z. ca* (*Ziphius cavirostris*); *M.bi* (*Mesoplodon bidens*); SEX (F = female, M = male); AGE (A = adult); SD (stranding date); SL (stranding location: T = Tenerife, F = Fuerteventura, L = Lanzarote, GC = Gran Canaria); SS (stranding stage: A = alive, D = dead); DC (decomposition stage: 1 = extremely fresh carcass, 2 = fresh carcass, 3 = moderate decomposition, 4 = advanced decomposition).

**Table 5:** Summary of the obtained sequences from *Ziphius cavirostris* in our study.

	* Case 3	Case 5	Case 8
CET	294	771	855
GenBank Acc. No.	GU066291	MZ066760	MZ066765
Samples	Lung and spleen	Brain	Lung
Nucleotide identity	100% MG437217 (*S. co*); KY680657 (*S.co*); KY680656 (*S. co*); KJ156331 (*S. co*)	98.48% KP995682 (*Z. ca*)	98.06% KP995682 (*Z. ca*)
Aminoacid identity	100% AUZ97325 (*S. co)* AUZ97326 (*S. co*) AHN91834 (*S. co*)	98.48% ALP00298 (*Z. ca*)	98.53% ALP00298 (*Z. ca*)

Notes: (*): animal previously published. *S. co* (*Stenella coeruleoalba*); *Z. ca* (*Ziphius cavirostris*).

**Table 6:** Summary of the obtained sequences from *the Mesoplodon genus* in our study. Bold percentages indicate novel sequences.

	* Case 1	Case 2	Case 4	Case 6	Case 7
CET	243	259	379	824	852
GenBank Acc. No.	JN863234	MZ066758	MZ066759	(a) MZ066761 (b) MZ066762	(a) MZ066763 (b) MZ066764
Species	*M. densirostris*	*M. europaeus*	*M. bidens*	*M. densirostris*	*M. densirostris*
Samples	Lung and kidney	Kidney	Lung and kidney	(a) Liver, prescapular lymph node and kidney (b) Brain	(a) Lung (b) Kidney
Nucleotide identity	95.65% KP995684 (*S.co*)	**78.35%** KP995682 (*Z. ca*)	**89.32%** JN863234 (*M. de*)	(a) 93.59% JN863234 (*M. de*) (b) 97.04% KP995684 (*S. co*)	(a) **78.3%** KF155406 (*D. le*) (b) 97.46% KP995684 (*S.co*)
Amino acid identity	79.13% ANG08598 (*D. le*)	82.81% ALP00298 (*Z. ca*)	95.59% ALP00300 (*S.co*)	(a) 97.06% ALP00300 (*S.co*) (b) 97.06% ALP00300 (*S.co*)	(a) 72.73% ALP00292 (*Z. ca*) (b) 96.92% ALP00300 (*S.co*)

Notes: (*): animal previously published. *S. co* (*Stenella coeruleoalba*); *D. le* (*Delphinapterus leucas*); *Z. ca* (*Ziphius cavirostris*); *M. de* (*Mesoplodon densirostris*).

## Data Availability

All beaked whales sequences have been deposited in GenBank (accession numbers: MZ066758, MZ066759, MZ066760, MZ066761, MZ066762, MZ066763, MZ066764, and MZ066765).
